# Evaluation of a Plant-Based Infant Formula Containing Almonds and Buckwheat on Gut Microbiota Composition, Intestine Morphology, Metabolic and Immune Markers in a Neonatal Piglet Model

**DOI:** 10.3390/nu15020383

**Published:** 2023-01-12

**Authors:** Manoj Gurung, Fernanda Rosa, Brooke Yelvington, Nathan Terry, Quentin D. Read, Brian D. Piccolo, Becky Moody, Patricia Tripp, Hoy E. Pittman, Bobby L. Fay, Talyor J. Ross, James D. Sikes, Jessica B. Flowers, Renee Fox, Tanya LeRoith, Rachelanne Talatala, Fabiana Bar-Yoseph, Laxmi Yeruva

**Affiliations:** 1 USDA-ARS, South East Area, Arkansas Children’s Nutrition Center, Little Rock, AR 72202, USA; 2 School of Veterinary Medicine, Texas Tech University, Amarillo, TX 79409, USA; 3 USDA-ARS, Southeast Area, North Carolina State University, Raleigh, NC 27695, USA; 4 Technical Services Envigo, Teklad Diets, Madison, WI 53713, USA; 5 Department of Biomedical Sciences & Pathobiology, Virginia Tech, Blacksburg, VA 24061, USA; 6 Else Nutrition GH, Tel Aviv-Yafo 6971070, Israel

**Keywords:** infant formula, plant-based, piglet model, metabolism, microbiota, neonates

## Abstract

A controlled-neonatal piglet trial was conducted to evaluate the impact of a plant-based infant formula containing buckwheat and almonds as the main source of protein compared to a commercially available dairy-based formula on the gut health parameters. Two day old piglets were fed either a plant-based or a dairy-based formula until day 21. Gut microbiome, cytokines, growth and metabolism related outcomes, and intestinal morphology were evaluated to determine the safety of the plant-based infant formula. This study reported that the plant-based formula-fed piglets had a similar intestinal microbiota composition relative to the dairy-based formula-fed group. However, differential abundance of specific microbiota species was detected within each diet group in the small and large intestinal regions and fecal samples. *Lactobacillus delbrueckii*, *Lactobacillus crispatus*, and *Fusobacterium* sp. had higher abundance in the small intestine of plant-based formula-fed piglets compared to the dairy-based group. *Bacteroides nordii*, *Enterococcus* sp., *Lactobacillus crispatus*, *Prevotella* sp., *Ruminococcus lactaris*, *Bacteroides nordii*, *Eisenbergiella* sp., *Lactobacillus crispatus*, *Prevotella* sp., and *Akkermansia muciniphila* had greater abundance in the large intestine of the plant based diet fed piglets relative to the dairy-based diet group. In the feces, *Clostridiales*, *Bacteroides uniformis*, *Butyricimonasvirosa*, *Cloacibacillus porcorum*, *Clostridium clostridioforme*, and *Fusobacterium* sp. were abundant in dairy-based group relative to the plant-based group*. Lachnospiraceae*, *Clostridium scindens*, *Lactobacillus coleohominis*, and *Prevetolla* sp. had greater abundance in the feces of the plant-based group in comparison to the dairy-based group. Gut morphology was similar between the plant and the dairy-based formula-fed piglets. Circulatory cytokines, magnesium, triiodothyronine (T3), thyroxine (T4), thyroid stimulating hormone (TSH), vitamin D, vitamin K, and IgE levels were similar among all piglets independent of dietary group. Overall, the present study demonstrated that a plant-based formula with buckwheat and almonds as the primary source of protein can support similar gut microbiota growth and health outcomes compared to a dairy-based infant formula.

## 1. Introduction

Diet is one of the most common factors that affects the microbiota composition [1,2]. Change in microbiota profile is often considered to impact the health status and has implications in human development [3,4]. In newborns, the gastrointestinal tract (GIT) undergoes rapid developmental changes during early life. These changes are the result of a complex interaction between diet, microbiota, and the host [5]. Adequate nutrient intake is also needed to maintain immune homeostasis, thus neonatal diets can significantly affect the immune system development in infants [6]. Hence, the evaluation of changes in the gut environment in terms of microbiota, morphology, and immunological changes is critical to validate the safety of nutritional compounds in neonates. In this scenario, alternatives to dairy-based infant formula are highly sought out due to several factors including lactose intolerance and animal milk allergy. Cow milk is the main protein source in dairy-based formulas and allergy to cow milk protein is common in infants [7,8,9,10]. Approximately 2 to 7.5% of children suffer from cow milk protein allergy during their first year of life, which is manifested by allergic symptoms in the skin, gastrointestinal tract, and respiratory tract [11,12]. Along with this reason, parents are seeking to feed infants and young children plant-based “milk” alternatives for additional reasons such as intolerances, or the perception that these conditions are present, or from reasons related to health beliefs or cultural values [13].

Plant-based formulas are one of the alternatives available where the protein source is replaced by a plant-based protein. In the United States, approximately 25% of plant-based formulas are soy based [12,14]. However, there are some concerns regarding soy formula due to its potential effects on sexual development and reproduction, neurobehavioral development, immune function, and thyroid function [14,15,16]. Hence, our previous study evaluated the safety of a plant-based formula containing almonds and buckwheat in a neonatal piglet model [17]. That study showed that both plant-based and dairy-based formula-fed groups had similar body weight gain and calorie intake, and the hematological parameters were within the reference value. There was no difference in organ development between the two diet groups. Liver function biomarkers, bilirubin, and alkaline phosphatase were higher in the plant-based formula-fed piglets at weeks 2 and 1, respectively. Plasma calcium levels were higher in the plant-based formula-fed piglets at week 2, whereas plasma glucose was lower in the plant-based formula group relative to the dairy-based fed piglets at 2 weeks of age [17]. These findings indicate that plant-based formula is safer compared to a dairy-based formula, and may also provide health benefits compared to dairy-based milk formula.

To further explore the health benefits as well as the safety of a plant-based infant formula (containing almonds and buckwheat as the primary source), the gut microbiota composition, systemic immune response, growth and metabolism related parameters, and intestinal morphology were evaluated. 

## 2. Materials and Methods

### 2.1. Experimental Design and Animal Care

The animal study was approved by the Institutional Animal Care and Use Committee (IACUC) at the University of Arkansas for Medical Sciences (UAMS IACUC protocol #4070). The detailed study design and diet composition were previously described [17]. Briefly, on postnatal day 2 (PND 2), crossbred (Yorkshire/Landrace cross with some Duroc on the bar side) piglets were purchased from a Class “A” licensed biomedical research farm (Oak Hill Genetics, Ewing, IL, USA) and were individually housed at the animal facility at the Arkansas Children’s Research Institute. Upon arrival, intact male and female piglets (*n* = 9 per group) were randomly assigned into either dairy-based formula (Similac Advance powder, Abbot Nutrition Laboratories, Columbus, OH, USA) or plant-based formula groups (Else Nutrition GH). Else Nutrition GH Ltd. provided the plant-based formula (main ingredients: almond butter and buckwheat flour) used in this study. The dairy-based formula (Similac Advance powder) was purchased from the commercial company (Abbott Nutrition Abbot Laboratories, Columbus, OH, USA). Supplementary nutrients formulated and produced by Envigo Teklad Diets (Madison, WI, USA) were added into both diets to meet the nutritional requirements of growing piglets by the National Research Council (NRC) [18]. Piglets were trained to nipple drink on a fixed schedule to provide 1.047 MJ/kg/day. Piglets were observed daily for any signs of discomfort, distress, and malabsorption.

### 2.2. Sample Collection

Blood samples were collected weekly, 2 h after morning feeding, starting at day 5 until 21 days of age. Prior to blood collection, piglets were anesthetized with a tight-fitting mask infused with 3–5% isoflurane and oxygen between 0.8 and 1.5 L/min. The neck was shaved and cleaned with 70% ethanol and blood was collected via cardiac puncture with an 18-gauge 3-inch needle and 20 mL syringes. Fecal samples were collected through the study period. On day 21, terminal bleeding was performed along with the collection of GIT contents. GIT contents were separated into six sections: duodenum, jejunum, ileum, cecum, proximal colon (PC), and distal colon (DC). The duodenal contents were collected 50 cm from the proximal end of the stomach. Ileal contents and jejunal contents were collected 50 cm and 229 cm from the distal end of the small intestine, respectively. DC contents were collected 50 cm from the end of the colon, and PC contents were obtained 25 cm from the cecum. For the cecum contents, a 10-cm section from the middle was utilized. Luminal contents were immediately snap-frozen in liquid nitrogen upon collection, and stored at −80 °C until use.

### 2.3. Microbiome Library Preparation, Sequencing, and Analysis

Fecal and intestinal contents from different regions were processed for microbiota data collection and analyses. DNA isolation, 16S rRNA library preparation, and sequencing were carried out by RTL Genomics (Lubbock, TX, USA). DNA was isolated using the Xymo ZR-96 Magbead Extraction Kit (ZYMO Research, cat#D4302, D4306, D4308) with the KingFisher FLEX instrument (ThermoFisher Scientific, Waltham, MA, USA) with some modifications. In brief, fecal samples were added into the ZR BashingBead Module and mechanically lysed with the Qiagen TissueLyser (Qiagen, Cat # 85300). Lysed samples were then transferred into 96 deep-well block for purification. V3–V4 16s rRNA regions were amplified for sequencing in a two-step process. The forward primer was constructed (5′–3′) with the forward Illumina overhang adapter (TCGTCGGCAGCGTCAGATGTGTATAAGAGACAG) added to the CTACGGGNGGCWGCAG primer. The reverse primer was constructed (5′–3′) with the reverse Illumina overhang adapter (GTCTCGTGGGCTCGGAGATGTGTATAAGAGACAG) added to the GACTACHVGGGTATCTAATCC primer. Amplifications were performed in 25 µL reactions with the Qiagen HotStar Taq master mix (Qiagen Inc, Valencia, CA, USA), 1 µL of each 5 µM primer, and 1 µL of template. Reactions were performed on ABI Veriti thermocyclers (Applied Biosytems, Carlsbad, CA, USA) under the following thermal profile: 95 °C for 5 min, then 35 cycles of 94 °C for 30 s, 54 °C for 40 s, 72 °C for 1 min, followed by one cycle of 72 °C for 10 min, and 4 °C hold. Products from the first stage amplification were added to a second PCR based on qualitatively determined concentrations. Primers for the second PCR were designed based on the Illumina Nextera PCR primers as follows: Forward-ATGATACGGCGACCACCGAGATCTACAC [i5index] TCGTCGGCAGCGTC and Reverse-CAAGCAGAAGACGGCATACGAGAT [i7index] GTCTCGTGGGCTCGG. The second stage amplification was run the same as the first stage except for 10 cycles.

Amplification products were visualized with eGels (Life Technologies, Grand Island, NY, USA). Products were then pooled equimolarly and each pool was size selected in two rounds using the SPRIselect Reagent (BeckmanCoulter, Indianapolis, Indiana) in a 0.75 ratio for both rounds. Size selected pools were then quantified using the Qubit 4 Fluorometer (Life Technologies) and loaded on an Illumina MiSeq (Illumina, Inc., San Diego, CA, USA).

The 16S rRNA sequencing data were analyzed using the RTL Genomics analysis pipeline (http://www.rtlgenomics.com/docs/Data_Analysis_Methodology.pdf, last updated 15 April 2019). Using this pipeline, short sequences, singleton sequences, and noisy reads were removed. Chimeras were removed using UCHIME chimera detection software executed in de novo mode. The remaining sequences were then clustered into operational taxonomic units (OTUs) using the UPARSE algorithm. The sequence from each cluster was then run using the USEARCH global alignment algorithm mode, requiring a 90% identity over the alignment against a database of high-quality sequences derived from the NCBI database. The output was then analyzed using the python program developed by RTL Genomics, which assigns taxonomic information to each sequence and then computes and writes the final analysis files.

### 2.4. Gastrointestinal Tract Histomorphometric Analyses

For histomorphometric analyses, small and large intestinal tissues were cut, the contents were flushed by formalin, fixed in formalin, embedded in paraffin, and processed to stain with hematoxylin and eosin (H and E). Small intestinal crypt depth, villi height, length, and width of Peyer’s patches, and gland depth of the large intestine and cecum were evaluated using Aperio Image Software by a board-certified pathologist (Dr. Tanya LeRoith). Intact crypts that were close to the measured villi and within the plane of section were selected for measurements.

### 2.5. Plasma Cytokines Measurements

Plasma cytokines were measured using porcine cytokine antibody array A (Abcam, cat#ab197479) targeting 10 cytokines (interleukins [IL]-1β, IL-4, IL-6, IL-8, IL-10, IL-12, GM-CSF, IFN-γ, TGF-β1, and TNF-α) as per the manufacturer’s protocol. In brief, 100 µL plasma samples were incubated in the cytokine antibody array for 1.5 h with gentle shaking and washed seven times using the provided wash buffer. The detection antibody cocktail was added into each well, incubated for 1 h at room temperature with gentle shaking, and followed by washing steps. In each well, Cy3 equivalent dye-conjugated streptavidin was added, and incubated at room temperature in the dark for 1 h. Then, the slide was washed, completely dried, and signals were visualized using an Innoscan 710 Microarray Scanner (INNOPSYS INC) with 532 nM acquisition mode, 50 µM pixel size, and 15× detection gain settings.

### 2.6. ELISA

Serum (day 21) vitamin D (Assaygenie, cat#HVD3), vitamin K (Assaygenie, cat#VK1), magnesium (Assaygenie, cat#MAES0123), T3 (Assaygenie, cat#PRFI00235), T4 (Assaygenie, cat#PRFI00228), TSH (Assaygenie, cat#PRFI00223), and IgE (Assaygenie, cat#PRFI00225) were measured using ELISA as per the manufacturer’s instructions.

### 2.7. Statistical Analysis

Microbiome data analyses were conducted using R software (v4.1.0.). The *p*-values were adjusted for false discovery correction using the Benjamini–Hochberg (BH) method [19] and considered significant at adjusted *p* < 0.05. OTUs were required to have a phylum classification to be considered informational. Beta diversity was estimated using the Bray–Curtis dissimilarity statistic. The dissimilarities were visualized using a 2D principal coordinate analysis (PCoA) plot and tested for differences between diets per region using a permutational multivariate analysis of variance (PERMANOVA) test with 999 permutations. The group dispersions were tested for homogeneity using an analysis of variance (ANOVA) test. Alpha diversity was estimated using the following metrics: Chao1, Faith’s PD, Simpson, Inverse Simpson, Observed OTUs, and Shannon. A Wilcoxon rank sum test (i.e., Mann–Whitney U) was used to compare these metrics between the diets for each region. Differential abundance was assessed at the OTU level for content data using DESeq2 [20], whereas linear mixed models were utilized for fecal repeated measures data. OTUs with <5 counts in 40% of the samples per region were removed prior to differential analysis. The differences in the phyla counts between the diets for each region were assessed using a Wilcoxon rank sum test (i.e., Mann–Whitney U). Assessment of sex was also included in each step of the analysis, but was found to be not significant. Histomorphometric parameters between formula treatments were compared using a repeated-measures linear mixed model with random intercepts for group and individual nested within group (*n* = 10 spatially separated measurements per individual); ANOVA was performed using the Satterthwaite approximation for the denominator degrees of freedom. Cytokine and ELISA data were analyzed by using GraphPad Prism version 9.3.1. Outliers were removed using ROUT (Q = 5%) and group comparisons were conducted using multiple Mann–Whitney tests.

## 3. Results

### 3.1. Plant-Based Formula Altered Microbiota Composition in the Gastrointestinal Tract

To elucidate how the plant-based diet impacted the gut microbiota of different gastrointestinal regions at different time points, 16S rRNA sequencing was performed in the small and large intestinal regions as well as in fecal samples at 6, 9, and 13 days of age.

**Figure 1:**
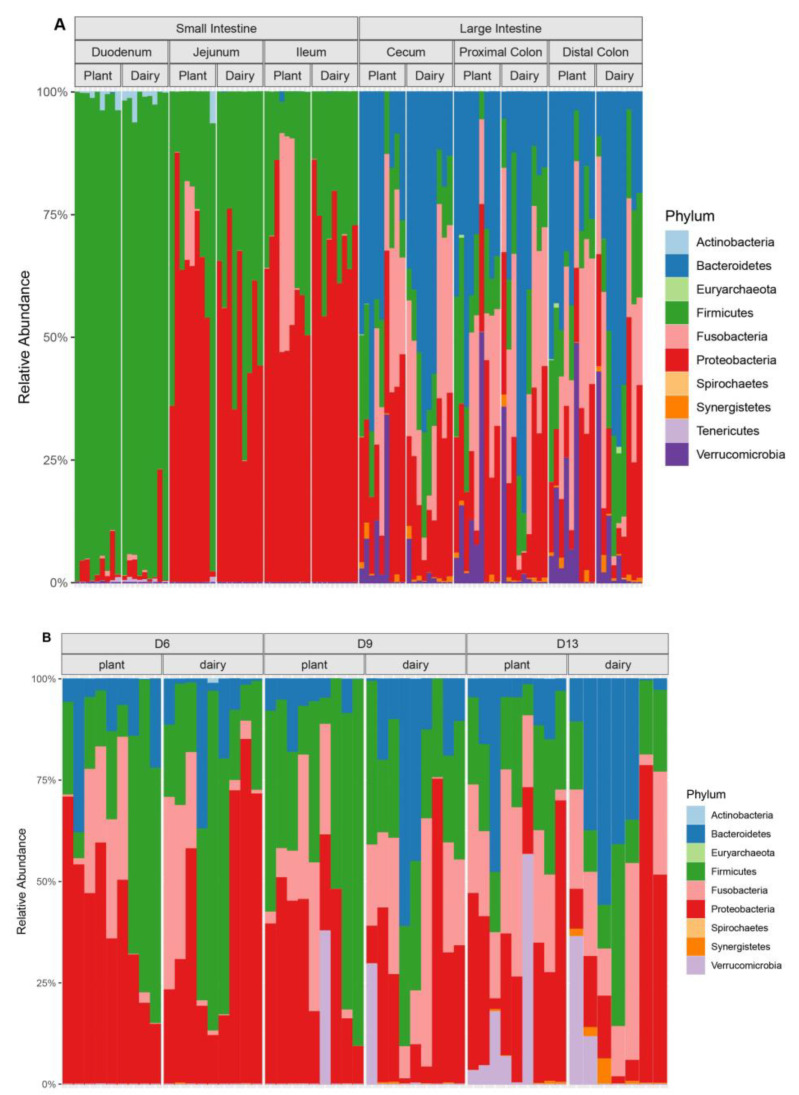
Distribution of the bacterial phyla within the small and large intestinal regions and feces of piglets fed either a plant- or a dairy-based formula diet. (**A**) Bacterial phyla within the small and large intestinal contents of day 21 old piglets. (**B**) Fecal microbiota composition on different days at the phyla level. Data generated by 16S rRNA amplicon sequencing targeting the V3–V4 variable region. Sequencing counts (OTUs) were summed to the phylum level and then proportionally normalized.

At the phylum level, the main difference was between the locations of the microbiota: small vs. large intestine (Figure 1A). Overall, both diets showed similar microbiota composition at the phylum level. In duodenum, Firmicutes was the predominant phylum in both diet groups, whereas Proteobacteria was the major phylum detected in jejunum and ileum. In the large intestine, Bacteroidetes was the predominant phylum within the microbiota composition. At days 6 and 13, no differences at the phylum level were observed in the feces between the dairy- and plant-based diet groups (Figure 1B). However, the dairy-based diet fed piglets had greater abundance of Bacteroidetes and Synergistetes in feces relative to the plant-based diet group at 9 days of age (Figure 1B). Within each diet group, fecal microbiota analysis revealed that the phylum Synergistetes had greater abundance at 13 days of age compared to 6 or 9 days (Figure 1B), showing similar trends in both the dairy-based and plant-based groups.

### 3.2. Microbiota Diversity

**Figure 2:**
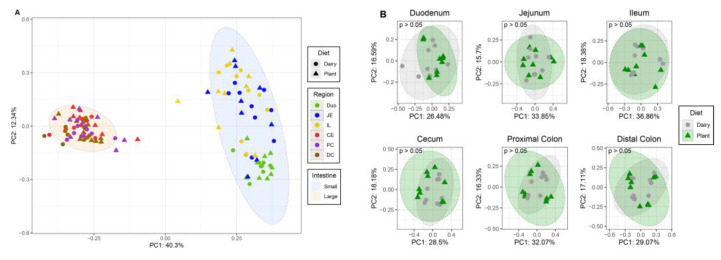
β-diversity in the intestinal contents of piglets fed either a plant- or a dairy-based formula diet. Data generated by 16S rRNA amplicon sequencing targeting the V3–V4 variable region. (**A**) Beta diversity in the small and large intestinal regions as estimated by sequencing counts (OTUs) using the Bray–Curtis dissimilarities and then visualized using principal coordinate analysis (PCoA). (**B**) β-diversity estimated on sequencing counts (OTUs) using Bray–Curtis dissimilarities and then visualized using principal coordinate analysis (PCoA). Diet difference in the beta diversity estimate was assessed using permutational multivariate analysis of variance (PERMANOVA). *n* = 9 per group. Duo—duodenum; JE—jejunum; IL—ileum; CE—cecum; PC—proximal colon; DC—distal colon.

**Figure 3:**
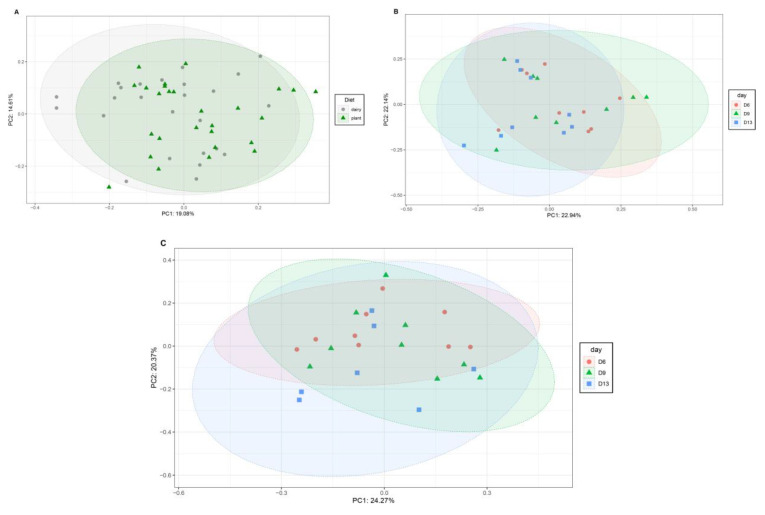
Fecal bacterial diversities between the plant-based and dairy-based formula-fed piglets. (**A**) Overall beta diversity of the fecal microbiota of 21-day old piglets estimated by sequencing counts (OTUs) using Bray–Curtis dissimilarities and then visualized using principal coordinate analysis (PCoA). *p* < 0.05, *r*^2^ = 0.05 (**B**) Fecal bacterial beta diversity in piglets fed a plant-based formula evaluated at 6, 9, and 13 days of age (*n* = 9/time point). Beta diversity was estimated by sequencing counts (OTUs) using Bray–Curtis dissimilarities and then visualized using principal coordinate analysis (PCoA). Permutational multivariate analysis of variance (PERMANOVA) was used to assess the effect of time. D13 vs. D6 (*p* < 0.05), D13 vs. D9 (*p* > 0.05), D6 vs. D9 (*p* > 0.05). (**C**) Fecal bacterial beta diversity in piglets fed a dairy-based formula at 6, 9, and 13 days of age (*n* = 9/time point). Beta diversity estimated by sequencing counts (OTUs) using Bray–Curtis dissimilarities and then visualized using principal coordinate analysis (PCoA). Permutational multivariate analysis of variance (PERMANOVA) was used to assess the effect of time. D13 vs. D6 (*p* < 0.05), D13 vs. D9 (*p* > 0.05), D6 vs. D9 (*p* > 0.05).

To assess the impact of plant or dairy-based diet on microbiota diversity, we calculated the beta and alpha diversities of microbiota from the large and small intestine regions as well as feces at different time points (Figure 2 and Figure 3). The Bray–Curtis dissimilarity showed differences in bacterial communities only between the large and small intestine (Figure 2A) within each diet. There was no significant difference in beta diversity between two diet groups in any regions of the intestine (Figure 2B).

In the feces, we observed a significant difference in the overall beta diversity (as measured by the Bray–Curtis dissimilarity, *p* < 0.05) between the dairy- and plant-based formula-fed piglets (Figure 3A). However, the *r*^2^ (i.e., explained variance) was less than 5%. Within each diet, beta diversity was different at 6 days compared to 13 days of age (Figure 3B,C). When a comparison between the two diets was performed at days 6, 9 and 13, there was no significant difference in beta diversity between the two groups (Supplementary Figures S1–S3).

We also calculated the alpha diversity, which measures the diversity within the samples (Supplementary Tables S1 and S2). There was no significant difference in the alpha diversity in the gastrointestinal regions and in the feces between the plant-based and dairy-based formula diet fed piglets. Among the calculated alpha diversity metrices, Chao1 index, Faith’s PD, Inverted Simpson, Observed OTUs, and Simpson measurements were highest in the proximal colon in both dietary groups.

### 3.3. Gut and Fecal Microbiota Composition at Taxonomic Level

**Figure 4:**
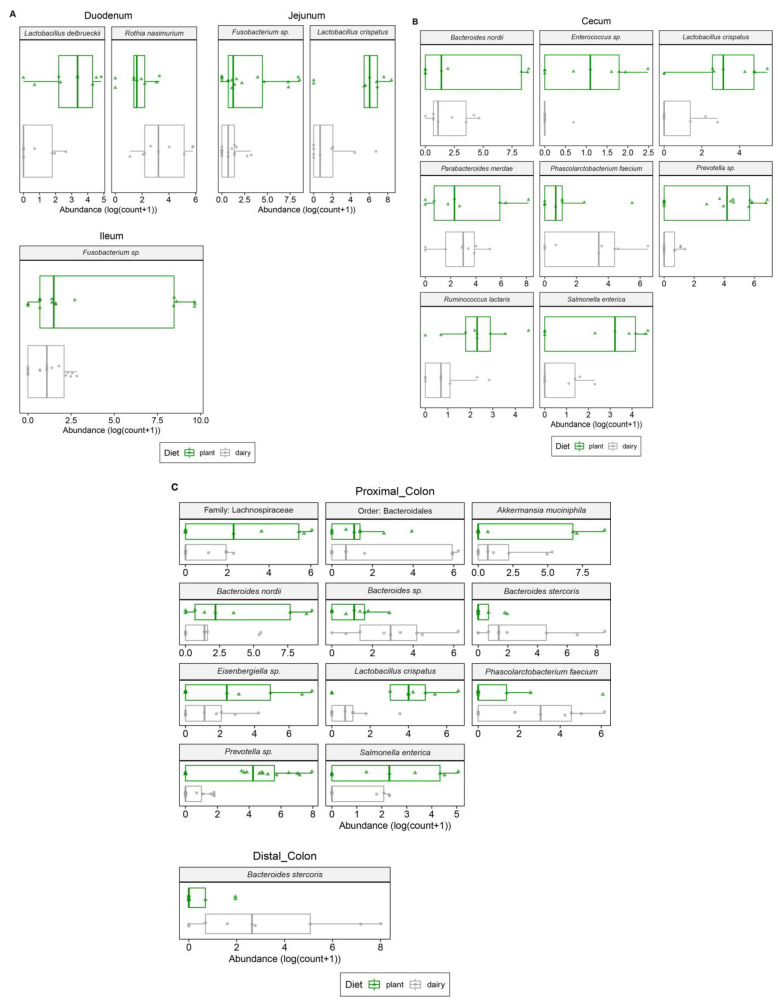
Differentially abundant bacteria in the intestinal contents from 21-day old piglets fed either a plant- or a dairy-based formula (*n* = 9 per group). Only the significantly abundant taxa are shown. (**A**) Differential abundance of sequencing counts (OTUs) of the small intestine contents was assessed using the DESeq2 pipeline. Significant differences were considered at *p* < 0.05 after correcting for false discovery correction using the BH method. Operational taxonomy units (OTUs) were considered if at least 20% of the samples contained a minimum of five counts. (**B**) Differential abundance of cecal content sequencing counts (OTUs) was assessed using the DESeq2 pipeline; significant differences were considered at *p* < 0.05 after correcting for false discovery correction using the BH method. OTUs were considered if at least 40% of samples contained a minimum of five counts. (**C**) Large intestinal contents microbial differential abundance of sequencing counts (OTUs) was assessed using the DESeq2 pipeline, significant differences were considered at *p* < 0.05 after correcting for false discovery correction using the BH method. OTUs were considered if at least 40% of the samples contained a minimum of five counts. All of the data shown were significant between diet groups.

Bacterial taxonomy was analyzed in the small and large intestinal regions of both the dairy- and plant-based formula diet fed piglets. In the small intestine, four bacterial taxa passed our statistical threshold (Figure 4A). *Lactobacillus delbrueckii* were abundant in the duodenum of the plant-based group while *Rothia nasimurium* was abundant in the dairy-based formula diet fed piglets (Figure 4A). In the jejunum, *Fusobacterium* sp. and *Lactobacillus crispatus* were abundant in the plant-based relative to the dairy-based formula diet fed piglets (Figure 4A), while *Fusobacterium* sp. had a higher abundance in the ileum of plant-based formula-fed piglets compared to the dairy-based group (Figure 4A).

In the large intestine, the significantly different bacterial taxa are shown in Figure 4B,C. *Bacteroides nordii*, *Enterococcus* sp., *Lactobacillus crispatus*, *Prevotella* sp., *Ruminococcus lactaris*, and *Salmonella enterica* had greater abundance in the cecum of the plant-based group compared to the dairy-based group (Figure 4B), while *Phascolarctobacterium faecium* were abundant in the dairy-based formula-fed group relative to the plant-based formula-fed in the cecum (Figure 4B). In the proximal colon, a bacterial family, Lachnospiraceae and a bacterial order, Bacteroidales, were abundant in the plant-based formula group relative to the dairy-based formula group. At the species level, *Bacteroides nordii*, *Eisenbergiella* sp., *Lactobacillus crispatus*, *Prevotella* sp., *Akkermansia muciniphila*, and *Salmonella enterica* had greater abundance in the proximal colon of the plant-based diet fed piglets relative to the dairy-based diet group (Figure 4C). Even though *Salmonella enterica* species were observed in the plant based group, Salmonella sp. was 0.14% (Supplementary Table S3). The lowest percentage of these species observed is likely to be due to the low level of contamination from the diet. However, as published previously from the same study, no diarrhea or any health issues were noted.

**Figure 5:**
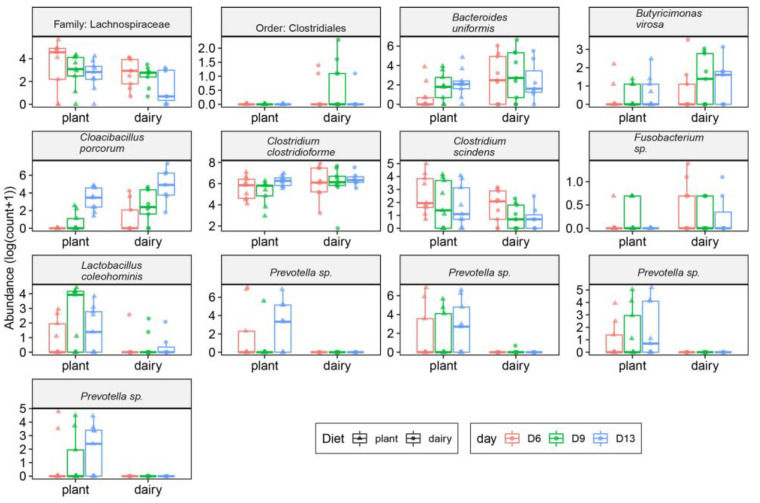
Differentially abundant bacteria in the feces collected at days 6, 9, and 13 of age in piglets (*n* = 9 per group) fed either a plant- or a dairy-based formula diet. Data generated by 16S rRNA amplicon sequencing targeting the V3–V4 variable region. Differential abundance of sequencing counts (OTUs) was assessed using linear mixed models. Significant differences were considered at *p* < 0.05 after correcting for false discovery correction using the BH method. All the data shown were significant between diet groups. Operational taxonomy units (OTUs) were considered if at least 40% of samples contained a minimum of five counts. Only the significantly abundant taxa are shown.

Fecal microbiota taxa from dairy- and plant-based fed piglets at 6, 9, and 13 days of age were also evaluated. A total of 13 OTUs were found to be significantly different between the plant- and dairy-based diet groups (Figure 5). *Clostridiales*, *Bacteroides uniformis*, *Butyricimonas virosa*, *Cloacibacillus porcorum*, *Clostridium clostridioforme*, and *Fusobacterium* sp. were abundant in the feces of the dairy-based formula diet fed piglets compared to the plant-based group, whereas *Lachnospiraceae*, *Clostridium scindens*, *Lactobacillus coleohominis*, and *Prevetolla* sp. had greater abundance in the feces of the plant-based group relative to the dairy-based group.

### 3.4. Plant-Based Infant Formula Impact on Gut Morphology

**Figure 6:**
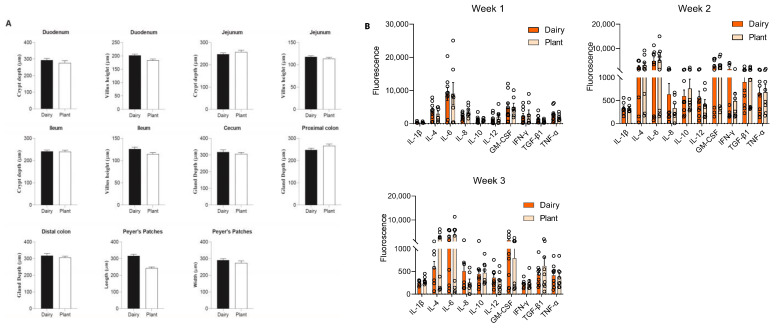
Histomorphometric analyses and plasma cytokines levels of piglets fed a dairy-based formula or a plant-based formula. (**A**) Histomorphometric parameters of duodenum, jejunum, ileum, cecum, colon, and Peyer’s patches. Histomorphometric parameters between formula treatments were compared using a repeated-measures linear mixed model with random intercepts for group and individual nested within group (*n* = 10 spatially separated measurements per piglet and *n* = 9/group); ANOVA was performed using the Satterthwaite approximation for the denominator degrees of freedom. *p* < 0.05 was considered significant. (**B**) Plasma cytokine levels at 1, 2, and 3 weeks of age of the piglets fed either a dairy-based or a plant-based formula (*n* = 9/group). Group differences were calculated using the Mann–Whitney test and *p* < 0.05 was considered significant.

To evaluate the impact of plant-based formula in the gastrointestinal tract, we performed histomorphometric analyses of Peyer’s patches, small intestine villi, and crypts, and large intestine gland length. The length of Peyer’s patches was smaller in the plant-based formula-fed piglets compared to the dairy-based group. However, after FDR correction, it was no longer significant. The width of Peyer’s patches, villi, and crypts of the small intestine, and the gland length of the cecum and large intestinal tissues were similar between the plant- and dairy-based fed piglets (Figure 6A, Supplementary Figure S4).

### 3.5. Plant-Based Formula and Its Effects on Circulatory Cytokines

To evaluate the effects of the plant-based relative to the dairy-based formula on the systemic immune response, plasma cytokines including IL-1β, IL-4, IL-6, IL-12, GM-CSF, TNF-α, IFN-γ, IL-8, IL-10, and TGF-β1 were measured in all piglets. The levels of cytokines are reported as the fluorescence signal. All the pro- and anti-inflammatory cytokines measured in the plasma of the piglets showed no statistical difference between the dairy-based and plant-based formula-fed groups (Figure 6B).

### 3.6. Plant-Based Infant Formula Impact on Growth, Metabolism Related Parameters, and Immunoglobulin E

To assess the impact of plant-based infant formula on the growth and metabolism of piglets, we measured the serum vitamin D, vitamin K, magnesium, triiodothronine (T3), thyroxine (T4), and thyroid stimulating hormone (TSH) at day 21 using ELISA. There was no difference between the two formula-fed groups (Supplementary Figure S5). Similarly, we also measured the immunoglobulin E (IgE), a major marker of allergic reaction. We observed no significant difference between the two groups (Supplementary Figure S5)

## 4. Discussion

Breastfeeding is the gold-standard source of nutrients to infants with proven health benefits, providing critical immune protection and shaping the microbiota composition [21,22,23]. However, due to several factors, infants receive formula diets in their early life when they are not exclusively breastfed. In fact, the majority of infants in North America receive infant formula diets by 2 months of age [14]. Although dairy-based milk formulas are the most used alternatives to human milk, plant-based formulas can be a healthy alternative to infants and might be a dietary source to avoid cow-milk and soy-protein allergic reactions. Therefore, in this study, we determined the effects of a plant-based formula containing almond and buckwheat flour as the main ingredients on the gut microbiota composition, systemic immune response, gut morphology, and host physiological markers using a neonatal piglet model.

Dietary changes including the transition from human milk to infant formula feeding can be a stressful event early in the life of neonates. Furthermore, the development of the intestinal microbiota can be hampered, leading to gut microbiota dysbiosis and consequently, increasing the risk of enteric infections [24,25]. In the current study, 16S rRNA sequencing of the gut microbiota revealed that in the duodenal region, the major phylum was Firmicutes, whereas in the jejunum and ileum, Firmicutes and Proteobacteria were the main phyla detected in both the dairy- and plant-based diet groups. These findings are in line with previous reports that analyzed the gut microbial composition of healthy pigs [26]. Along with the intestinal microbiota profile, the diversity metrics, which are known to impact the overall health in children, were similar between both the dairy- and plant-based formulas in all of the gastrointestinal regions. Within the gut microbiota composition in this study, some potential probiotics species were higher in the plant-based formula-fed piglets compared to the dairy formula-fed group including *Lactobacillus delbrueckii* and *Lactobacillus cripsatus*. Lactobacillus is one of the core genera in the porcine gut microbiota with an average abundance of 11.0% of the total bacteria taxa at 10 weeks of age [27]. Oral administration of *Lactobacillus delbrueckii* has been reported to upregulate tight junction genes, increase anti-inflammatory cytokine (IL-10), and decrease pro-inflammatory cytokines in lipopolysaccharide challenged weaned piglets [28]. Interestingly, a mucin-degrading bacterium, *Akkermansia muciniphila*, was abundant in the proximal colon of the plant-based fed piglets relative to the dairy-based group [29]. This bacterium has been shown to improve glucose tolerance, reverse diet induced metabolic disorders, insulin sensitivity, and improve the colon mucosal barrier [30,31]. Our previous study also showed lower glucose level in the plasma of the plant-based fed piglets [17] compared to that of the dairy-based group. The plant-based formula-fed piglets had greater abundance of *Prevotella* sp., *Lactobacillus coleohominis*, and *Clostridium scindens* in their feces compared dairy-based formula-fed group. Indeed, *Prevotella* is one of the most abundant bacterial taxa in the gastrointestinal tract of pigs and it has been associated with growth performance, greater feed conversion, and improved protection against diarrhea [32]. Additionally, in weaned piglets, both *Prevotella* and *Lactobacillus* were the most abundant bacterial genera [33] and their increased abundance has been reported following introduction of a plant-based diet in weaned piglets [34]. The intestinal microbiota profiling of the dairy-based formula-fed piglets in this present study are in accordance with previous studies that demonstrated that nursing piglets had higher abundance of Bacteroides, which utilize milk oligosaccharides as substrates [33,34]. Postnatal conditions can also affect the gastrointestinal tract development [35,36]. Therefore, we analyzed the gut morphology of both large and small intestinal regions. We found no significant differences in any of the histomophological measurements between the two formula-fed groups.

Early life exposure to adequate nutrients supply is essential to maintain normal immune response [6]. Hence, we compared the systemic cytokine level between the plant-based formula and the established dairy-based formula tested in this study. However, the lack of statistical difference in the cytokines level in blood of all piglets indicates that the plant-based formula provide similar effects at the systemic level when compared to well-known dairy based infant formula. The present study also demonstrated that the plant-based and dairy based infant formula-fed piglets had similar levels of serum vitamin D, vitamin K, magnesium, T3, T4, and TSH, suggesting comparable growth and metabolism between the two groups. Likewise, a similar level of serum IgE between the two groups indicates that plant-based formula imparts a similar effect on the host. We noticed the abundance of some potential pathogenic bacteria such as *Salmonella enterica* in the plant-based formula group when compared to the dairy-based formula group, however, no detrimental health outcomes were observed in these piglets throughout the trial [17]. Furthermore, a similar systemic immune response was observed in both diet groups. Overall, these outcomes can be attributed to the increased relative abundance of potential beneficial microbiota such as *Prevotella* and *Lactobacillus* in the plant-based fed piglets, which could led to the maintenance of a balanced microbiota composition.

## 5. Limitations

OTU-based clustering was used to define the bacterial taxonomy, which may have a lower taxonomy resolution compared to amplicon sequence variants (ASVs) [37]. However, recent evidence also suggests bias in ASVs due to the propensities for ASVs to define separate ASVs for bacterial species containing multiple 16S genes that differ in nucleotide sequencing [38]. As we did not identify major changes in bacterial communities between the plant- and milk-based formula group, further taxonomy resolution was not necessary in this study.

## 6. Conclusions

In summary, the findings from the present study demonstrate that the plant-based formula had some differential effects on the piglets’ gut health compared to the dairy-based formula-fed piglets. The plant-based formula promoted an intestinal microbiota composition similar to the well-characterized gut microbiota profile of weaned healthy piglets. This study also showed that both diet groups had a similar effect on the systemic immune response, growth, and metabolism associated parameters in the neonatal piglets. Long-term effects of the plant-based formula are yet to be determined as the current model recapitulates 3 month old infants.

## Data Availability

Accession no. to access the 16S microbiota data: BioProject ID PRJNA878504.

## Supplementary Materials

The following are available online at https://www.mdpi.com/article/10.3390/nu15020383/s1, Table S1: Alpha diversity measured in different intestinal regions of the piglets at day 21; Table S2: Alpha diversity measured in different intestinal regions of the piglets at days 6, 9 and 13; Table S3: *S. enterica* species’ relative abundance in the large intestine; Figure S1: Bacterial communities in the feces at postnatal day 6 in piglets (N = 18, 9/group) fed either a plant- or a dairy-based formula diet; Figure S2: Bacterial communities in the feces at postnatal day 9 in piglets (N = 18, 9/group) fed either a plant- or a dairy-based formula diet; Figure S3: Bacterial communities in the feces at postnatal day 13 in piglets (N = 18, 9/group) fed either a plant- or a dairy-based formula diet; Figure S4: Representative H&E images of the small and large intestine of piglets fed either a plant- or a dairy-based formula diet at day 21; Figure S5: Growth and metabolism related parameters and immunoglobulin E in the serum of 21 day old piglets fed either a plant- or a dairy-based formula diet.
