# Supplementary material for: Evaluation of a Plant-Based Infant Formula Containing Almonds and Buckwheat on Gut Microbiota Composition, Intestine Morphology, Metabolic and Immune Markers in a Neonatal Piglet Model
Source: Nutrients. 2023 Jan 12;15(2):383. doi: 10.3390/nu15020383 (PMC9861483; doi:10.3390/nu15020383)
Supplement: Supplementary file 1 [file nutrients-15-00383-s001.zip › Supplementary Material MG.pdf]

Supplementary Figures Legend

Supplementary Figure S1: Bacterial communities in the feces at postnatal day 6 in piglets (n=18) fed either a plant- or a dairy-based formula diet. Data generated by 16S rRNA amplicon sequencing targeting the V3-V4 variable region. Beta-diversity estimated on sequencing counts using Bray-Curtis dissimilarities and then visualized using Principal Co-ordinate Analysis (PCoA).

Supplementary Figure S2: Bacterial communities in the feces at postnatal day 9 in piglets (n=18) fed either a plant- or a dairy-based formula diet. Data generated by 16S rRNA amplicon sequencing targeting the V3-V4 variable region. Beta-diversity estimated on sequencing counts using Bray-Curtis dissimilarities and then visualized using Principal Co-ordinate Analysis (PCoA).

Supplementary Figure S3: Bacterial communities in the feces at postnatal day 13 in piglets (n=18) fed either a plant- or a dairy-based formula diet. Data generated by 16S rRNA amplicon sequencing targeting the V3-V4 variable region. Beta-diversity estimated on sequencing counts using Bray-Curtis dissimilarities and then visualized using Principal Co-ordinate Analysis (PCoA).

Supplementary Figure S4: Histomorphometric analyses of piglets fed a dairy-based formula or a plant-based formula. (A) Histomorphometric parameters of duodenum, jejunum, ileum, cecum, colon and peyer's patches. Histomorphometric parameters between formula treatments were compared using a repeated-measures linear mixed model with random intercepts for group and individual nested within group (n = 10 spatially separated measurements per indi-vidual); ANOVA was performed using the Satterthwaite approximation for the denominator degrees of freedom. $P<0.05$ was considered significant.

Supplementary Figure S5: Growth and metabolism related parameters and Immunoglobulin E in the serum of day 21 piglet fed either a plant- or a dairy-based formula diet. Mann-Whitney test, $p <0.05$ considered significant. ns – not significant

Supplementary Table S1: Alpha diversity measured in different intestinal regions of piglets at day 21 which were fed either dairy-based or plant-based formula diet. Data generated by 16S rRNA amplicon sequencing targeting the V3-V4 variable region. A Wilcoxon rank sum test (i.e Mann-Whitney U) was used to compart these metrics between two diet groups.

Supplementary table S1: Alpha Diversity in different intestinal regions							
		Plant		Dairy			
Alpha Diversity	Region	Median	IQR	Median	IQR	FDR	Differences 95% CI
Chao1	Duodenum	156.4	(147.88, 173.31)	144.25	(136.4, 163.75)	0.9072	(-15.88, 33.23)
Chao1	Jejunum	171.05	(162.59, 192.07)	150.8	(138.67, 177.04)	0.9072	(-18.75, 43.32)
Chao1	Ileum	166.87	(146.07, 187.36)	163.06	(152.5, 171.07)	0.9072	(-27.35, 51.96)
Chao1	Cecum	213.44	(211, 246)	223.06	(200.14, 232.15)	1	(-23.65, 30)
Chao1	Proximal Colon	231.75	(208, 237.12)	227.24	(213.38, 239.44)	1	(-50.29, 32.32)
Chao1	Distal Colon	230.45	(175.11, 239.65)	206.18	(192.95, 224)	0.9072	(-34.07, 46.7)
FaithsPD	Duodenum	8	(7.11, 8.98)	8.32	(7.17, 8.57)	1	(-1.45, 1.63)
FaithsPD	Jejunum	6.75	(5.8, 8.02)	6.5	(5.32, 7.16)	0.9072	(-0.82, 2.31)
FaithsPD	Ileum	6.91	(5.58, 7.55)	6.63	(6.26, 6.8)	0.9072	(-1.13, 1.41)
FaithsPD	Cecum	12.03	(10.82, 12.46)	12.12	(10.97, 12.84)	0.9072	(-2.41, 1.34)
FaithsPD	Proximal Colon	12.93	(11.13, 13.46)	12.36	(11.56, 13.76)	0.9213	(-3.27, 1.6)
FaithsPD	Distal Colon	12.54	(9.51, 13.03)	11.56	(11.2, 14.31)	0.9072	(-2.35, 1.73)
InvSimpson	Duodenum	5.9	(3.48, 8.9)	6.63	(5.07, 9.77)	0.9072	(-4.09, 2.83)
InvSimpson	Jejunum	8.36	(5.55, 9.12)	5.51	(4.68, 7.63)	0.9072	(-1.51, 4.22)
InvSimpson	Ileum	6.28	(4.73, 11.93)	6.18	(3.45, 7.77)	0.9072	(-2.74, 5.33)
InvSimpson	Cecum	8.57	(8.12, 10.46)	9.4	(7.71, 12.02)	0.9072	(-3.59, 1.66)
InvSimpson	Proximal Colon	8.76	(8, 12)	10.56	(7.13, 12.19)	1	(-3.43, 2.69)
InvSimpson	Distal Colon	8.33	(7.3, 9.49)	9.64	(8.9, 14.77)	0.9072	(-7.14, 2.02)

Observed	Duodenum	138	(123, 156)	132	(114, 151)	0.9072	(-15, 31)
Observed	Jejunum	140	(124, 159)	134	(113, 167)	0.9213	(-27, 30)
Observed	Ileum	121	(114, 153)	135	(121, 145)	1	(-25, 36)
Observed	Cecum	185	(179, 203)	180	(161, 187)	1	(-29, 26)
Observed	Proximal Colon	201	(175, 202)	191	(179, 212)	1	(-47, 23)
Observed	Distal Colon	190	(146, 208)	177	(155, 198)	0.9213	(-31, 47)
Shannon	Duodenum	2.52	(1.86, 2.68)	2.67	(2.23, 2.78)	0.9072	(-0.59, 0.43)
Shannon	Jejunum	2.64	(2.21, 2.76)	2.15	(2.1, 2.65)	0.9072	(-0.16, 0.64)
Shannon	Ileum	2.41	(2.04, 3.06)	2.44	(1.95, 2.69)	0.9072	(-0.47, 0.66)
Shannon	Cecum	2.85	(2.57, 3.2)	2.83	(2.75, 3.31)	0.9072	(-0.45, 0.33)
Shannon	Proximal Colon	2.93	(2.76, 3.1)	2.95	(2.68, 3.06)	1	(-0.39, 0.33)
Shannon	Distal Colon	2.87	(2.34, 2.91)	3.01	(2.86, 3.44)	0.9072	(-0.68, 0.24)
Simpson	Duodenum	0.83	(0.71, 0.89)	0.85	(0.8, 0.9)	0.9072	(-0.14, 0.06)
Simpson	Jejunum	0.88	(0.82, 0.89)	0.82	(0.79, 0.87)	0.9072	(-0.04, 0.1)
Simpson	Ileum	0.84	(0.79, 0.92)	0.84	(0.71, 0.87)	0.9072	(-0.06, 0.11)
Simpson	Cecum	0.88	(0.88, 0.9)	0.89	(0.87, 0.92)	0.9072	(-0.04, 0.02)
Simpson	Proximal Colon	0.89	(0.87, 0.92)	0.91	(0.86, 0.92)	1	(-0.04, 0.03)
Simpson	Distal Colon	0.88	(0.86, 0.89)	0.9	(0.89, 0.93)	0.9072	(-0.07, 0.02)

Supplementary Table S2: Alpha diversity measured in different intestinal regions of piglets at day 6, 9 and 13 which were fed either dairy-based or plant-based formula diet. Data generated by 16S rRNA amplicon sequencing targeting the V3-V4 variable region. A Wilcoxon rank sum test (i.e Mann-Whitney U) was used to compart these metrics between two diet groups.

Supplementary Table S2: Alpha Diversity in fecal samples						
	Day 6					
Alpha Diversity	Plant Median	Plant IQR	Dairy Median	Dairy IQR	p-value	95% CI Diff between diets
Chao1	86	(76.33, 93.14)	79.57	(75.2, 91.33)	0.7066	(-25.71, 17.83)
FaithsPD	7.35	(6.6, 8.14)	7.77	(7.07, 8.54)	0.931	(-1.71, 1.57)
InvSimpson	3.89	(3.04, 5.46)	3.92	(2.54, 5.53)	0.8406	(-2.02, 2.45)
Observed	81	(71, 87)	77	(71, 84)	0.6525	(-23.73, 15.28)
Shannon	1.83	(1.66, 2.25)	1.95	(1.48, 2.58)	0.968	(-0.5, 0.52)
Simpson	0.74	(0.67, 0.82)	0.75	(0.61, 0.82)	0.8472	(-0.14, 0.12)
	Day 9					
Alpha Diversity	Plant Median	Plant IQR	Dairy Median	Dairy IQR	p-value	95% CI Diff between diets
Chao1	93.88	(70.43, 100.6)	97.5	(78.09, 119.56)	0.3355	(-11.35, 31.35)
FaithsPD	7.99	(7.5, 9.79)	9.5	(7.13, 10.58)	0.4826	(-1.26, 2.55)
InvSimpson	4.38	(3.87, 5.42)	6.67	(4.89, 9.57)	0.2381	(-1.23, 4.61)
Observed	91	(65, 95)	94	(71, 110)	0.4077	(-11.78, 27.56)
Shannon	2.22	(1.98, 2.31)	2.39	(2.01, 2.82)	0.5866	(-0.36, 0.62)
Simpson	0.77	(0.74, 0.82)	0.85	(0.8, 0.9)	0.8811	(-0.11, 0.13)
	Day 13					
Alpha Diversity	Plant Median	Plant IQR	Dairy Median	Dairy IQR	p-value	95% CI Diff between diets
Chao1	106.88	(97, 115.09)	99.5	(83.51, 105.21)	0.2048	(-34.92, 8.19)
FaithsPD	9.96	(8.3, 10.91)	8.94	(7.27, 9.04)	0.1626	(-3.52, 0.65)

InvSimpson	5.11	(3.9, 6.89)	4.96	(3.64, 5.29)	0.9666	(-2.99, 2.88)
Observed	100	(91, 109)	91	(77, 92.5)	0.098	(-35.81, 3.4)
Shannon	2.3	(2.1, 2.55)	2.2	(1.87, 2.27)	0.5082	(-0.67, 0.35)
Simpson	0.8	(0.74, 0.85)	0.8	(0.72, 0.81)	0.7001	(-0.18, 0.12)

Supplementary Table S3: S. enterica species relative abundance in the large intestine

Supplementary Table S3: S. enterica species relative abundance in the large intestine			
Region	diet	Species	relative_abundance (%)
Distal Colon	dairy	S. enterica	0.00%
Distal Colon	dairy	S. enterica	0.00%
Distal Colon	dairy	S. enterica	0.00%
Distal Colon	dairy	S. enterica	0.00%
Distal Colon	dairy	S. enterica	0.00%
Distal Colon	dairy	S. enterica	0.00%
Distal Colon	dairy	S. enterica	0.00%
Distal Colon	dairy	S. enterica	0.00%
Distal Colon	plant	S. enterica	0.07%
Distal Colon	plant	S. enterica	0.00%
Distal Colon	plant	S. enterica	0.00%
Distal Colon	plant	S. enterica	0.01%
Distal Colon	plant	S. enterica	0.00%
Distal Colon	plant	S. enterica	0.00%
Distal Colon	plant	S. enterica	0.00%
Distal Colon	plant	S. enterica	0.00%
Proximal Colon	dairy	S. enterica	0.01%
Proximal Colon	dairy	S. enterica	0.00%
Proximal Colon	dairy	S. enterica	0.00%
Proximal Colon	dairy	S. enterica	0.00%
Proximal Colon	dairy	S. enterica	0.00%
Proximal Colon	dairy	S. enterica	0.00%
Proximal Colon	dairy	S. enterica	0.00%
Proximal Colon	dairy	S. enterica	0.00%
Proximal Colon	plant	S. enterica	0.12%
Proximal Colon	plant	S. enterica	0.00%
Proximal Colon	plant	S. enterica	0.00%
Proximal Colon	plant	S. enterica	0.00%
Proximal Colon	plant	S. enterica	0.00%
Proximal Colon	plant	S. enterica	0.00%
Proximal Colon	plant	S. enterica	0.00%
Proximal Colon	plant	S. enterica	0.00%
