# Supplementary figures and images for: Evaluation of a Plant-Based Infant Formula Containing Almonds and Buckwheat on Gut Microbiota Composition, Intestine Morphology, Metabolic and Immune Markers in a Neonatal Piglet Model
Source: Nutrients. 2023 Jan 12;15(2):383. doi: 10.3390/nu15020383 (PMC9861483; doi:10.3390/nu15020383)

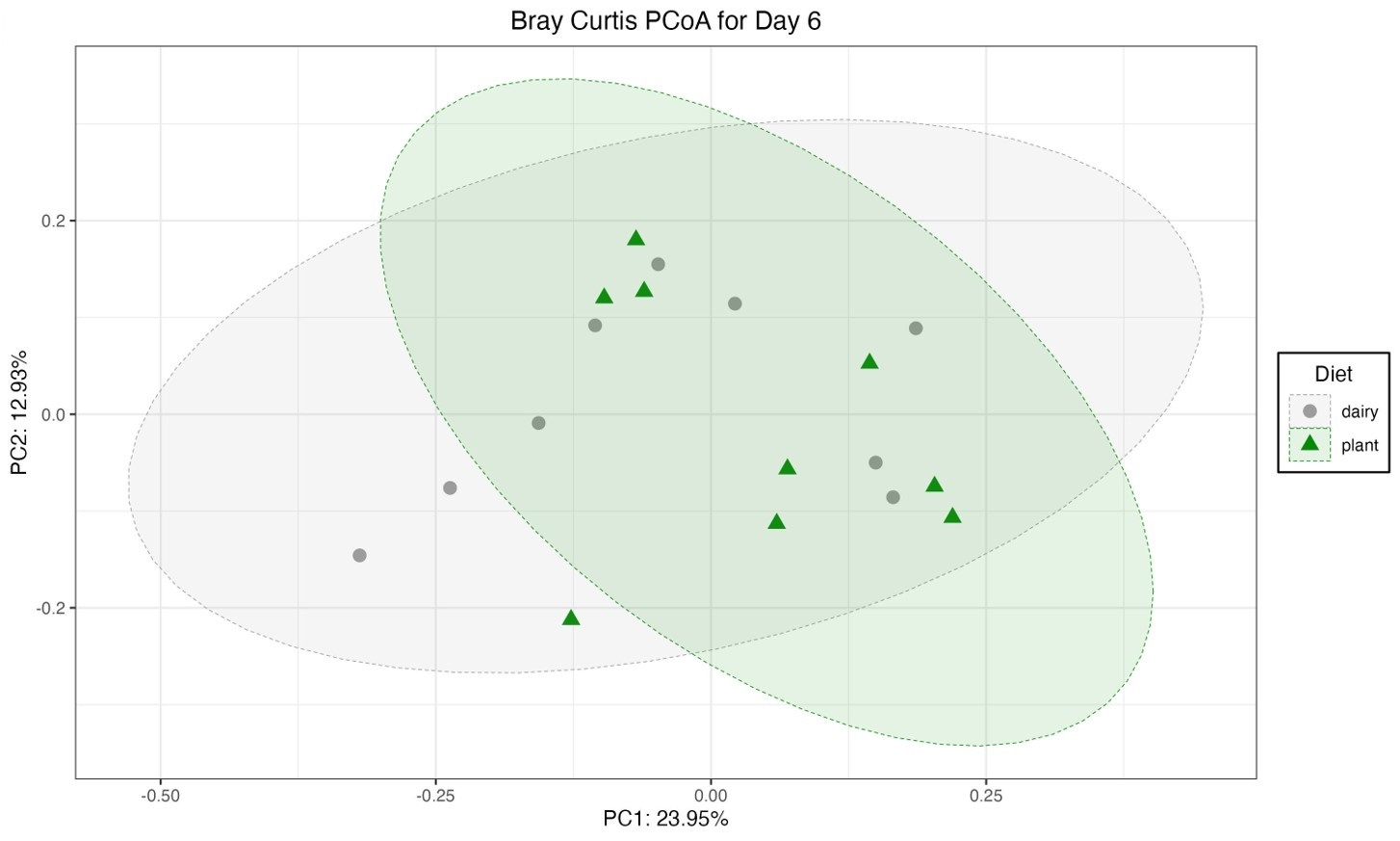

Supplement: Supplementary file 1 [file nutrients-15-00383-s001.zip › Figure S1.jpg]

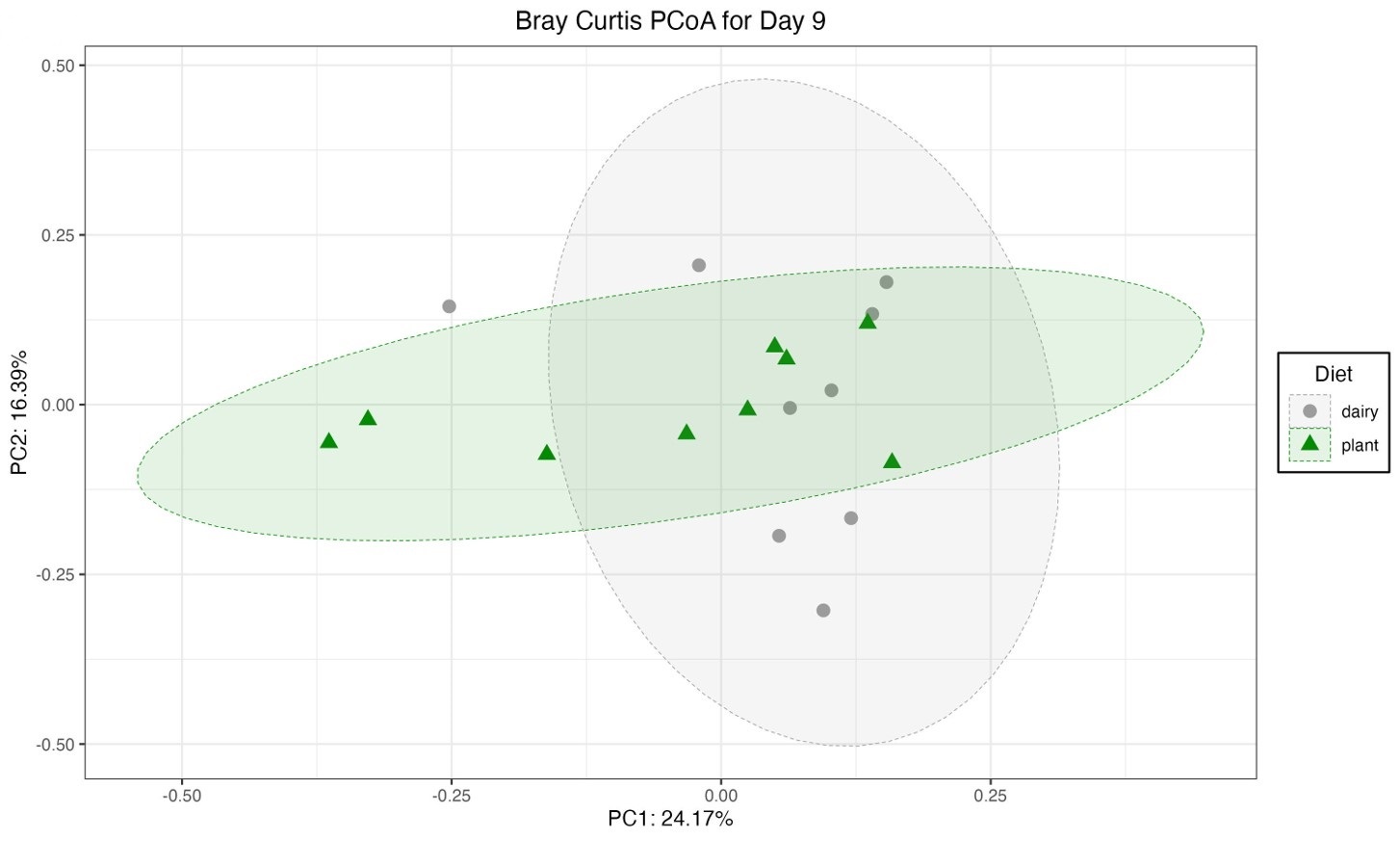

Supplement: Supplementary file 1 [file nutrients-15-00383-s001.zip › Figure S2.jpg]

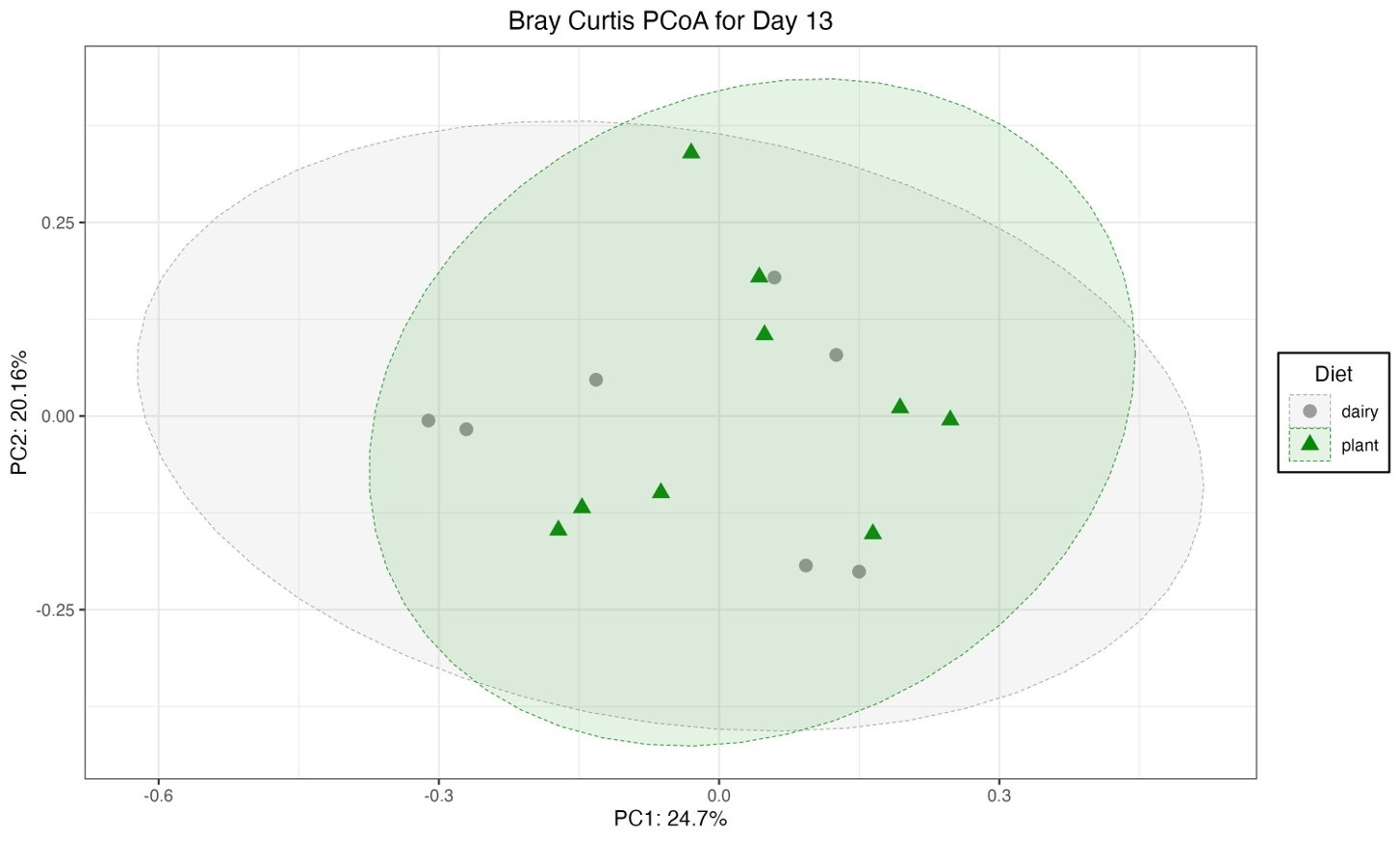

Supplement: Supplementary file 1 [file nutrients-15-00383-s001.zip › Figure S3.jpg]

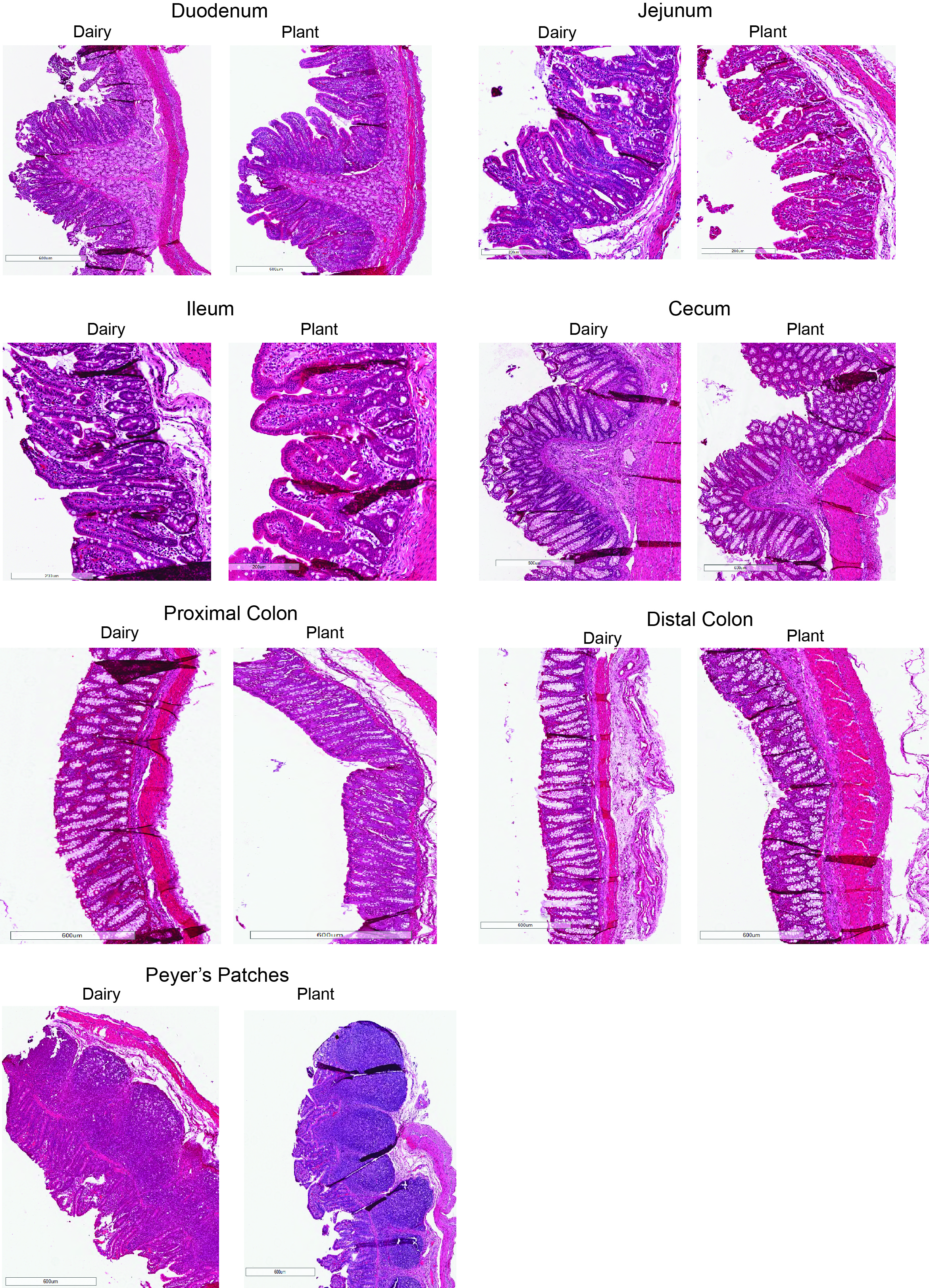

Supplement: Supplementary file 1 [file nutrients-15-00383-s001.zip › Figure S4.jpg]

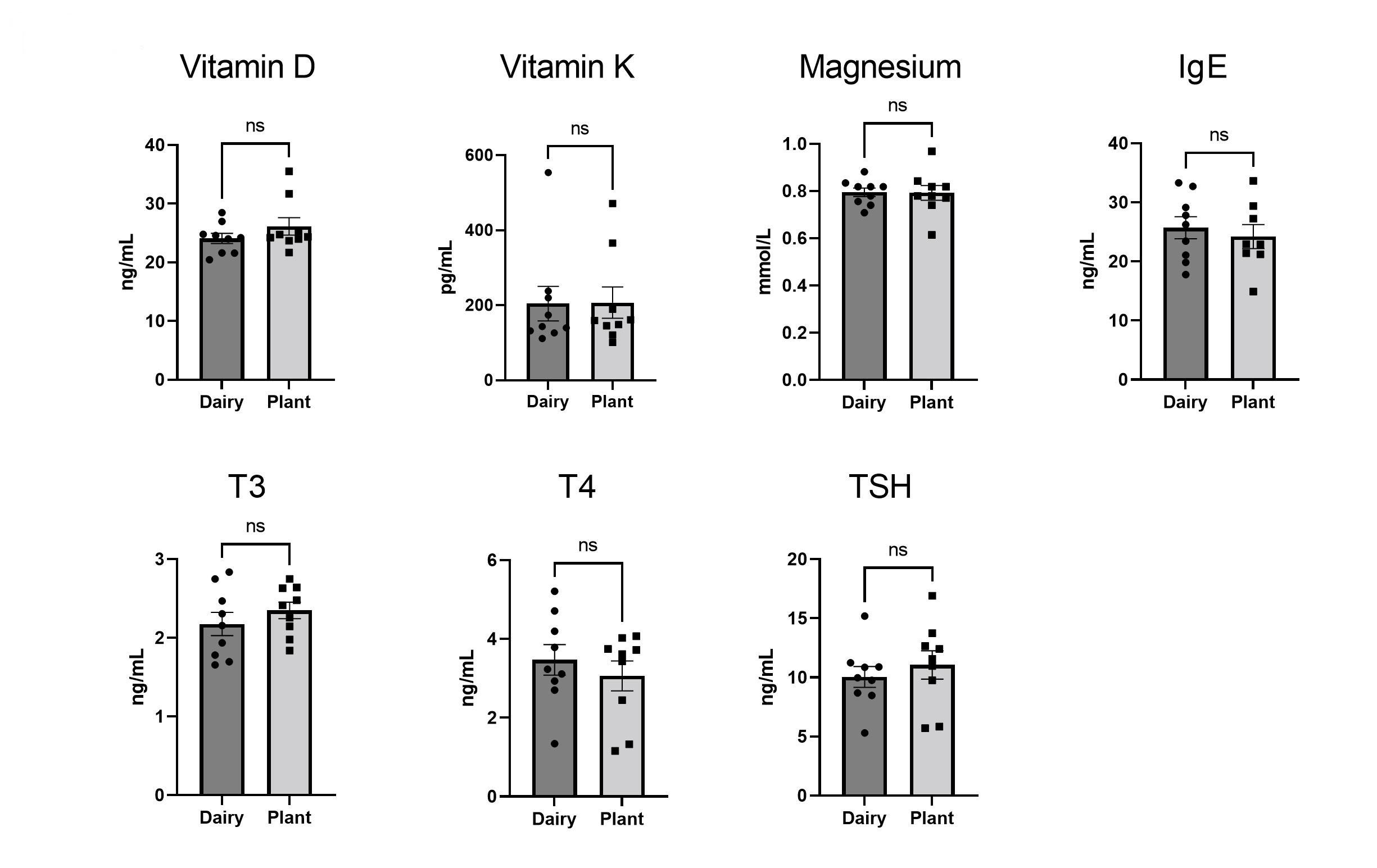

Supplement: Supplementary file 1 [file nutrients-15-00383-s001.zip › Figure S5.jpg]
